# Do trained nurses feel more psychologically safe?—Results from a multi‐level modelling approach

**DOI:** 10.1002/nop2.1015

**Published:** 2021-08-01

**Authors:** Melissa Seibert, Holger Pfaff, Nadine Scholten, Ludwig Kuntz

**Affiliations:** ^1^ Department for Business Administration and Health Care Management Faculty of Management, Economics and Social Sciences University of Cologne Germany; ^2^ Institute of Medical Sociology, Health Services Research, and Rehabilitation Science Faculty of Human Sciences and Faculty of Medicine University of Cologne Germany

**Keywords:** additional training, multilevel modelling, neonatal intensive care, neonatal intensive care unit, nursing, professional education, psychological safety, team structure

## Abstract

**Aim:**

To analyse the associations between a nurse's psychological safety and her/his additional training.

**Design:**

A cross‐sectional survey conducted between September 2015 and August 2016.

**Methods:**

A multi‐level modelling approach was used considering unit membership. We used data from 1,239 questionnaires completed by nurses on 75 different German neonatal intensive care units, and 75 questionnaires completed by the corresponding leading nurse.

**Results:**

We found the additional *managerial* training as a charge nurse to be a positive predictor for psychological safety (β = .346, *p* ≤ .05). Surprisingly, the additional *clinical* training in paediatric intensive care is negatively associated with psychological safety (β = −.192, *p* ≤ .01). Our model estimates that this negative association can be inhibited if the team's share of nurses with an additional clinical training increases (β = .313, *p* ≤ .05).

## 1 INTRODUCTION

Patient safety is related, amongst other things, to the behaviour and attitudes of nurses (Ridelberg et al., 2014). Nurses often have to decide spontaneously whether to raise their concerns in routine care, for example with regard to a particular treatment, and thus questioning traditional practices or their superiors. If nurses are afraid that their statements may have negative consequences for themselves and therefore do not address potential shortcomings in care, this restraint jeopardizes patient safety (Rabøl et al., 2011). Nurses feel psychologically safe, if they are confident that the team will not punish them for speaking up (Edmondson, 1999); “speaking up” is defined in this context as the “communication of ideas, suggestions, concerns or opinions about work‐related issues with the intent to improve organizational or unit functioning” (Morrison, 2011). Therefore, it is becoming increasingly important for hospitals to promote psychological safety amongst nurses and to use it to identify and remedy shortcomings in patient safety at an early stage. The importance of psychological safety for safe health‐care organizations has recently been highlighted by the National Health Service in England (NHS England, 2019); also, global corporations like Google declare psychological safety to be their first of five key dynamics for teams to work effectively and successfully (Rozovsky, 2015).

In general, it is assumed that patient safety could be improved by a high level of education on the part of the caring nurses. It is suggested that the higher the level of education is, the higher the patient safety and the quality of care is (Aiken et al., 2012; Kirwan et al., 2013). Staffing guidelines even require (a certain share of) nurses to possess additional training (for example, American Academy of Pediatrics Committee on Fetus and Newborn and American College of Obstetricians and Gynecologists Committee on Obstetric Practice 2017; Federal Joint Comitee 2020). In Germany, nurses complete three years of vocational training. After their vocational training and at least two years of professional experience, they might complete further additional training, for example in the field of paediatric intensive care. Within these advanced training courses, nurses deepen and broaden their theoretical knowledge and learn special forms of nursing care in a practical way. However, it is still uninvestigated whether these nurses with additional training also feel more psychologically safe and are able to apply their knowledge by speaking up even in critical situations. We will focus on the association between a nurse's additional training and her/his perceived psychological safety.

## 2 BACKGROUND

The idea of psychological safety was first introduced by Schein and Bennis in 1965, and described similarly by Kahn in 1990. Respectively, they describe it as a climate that tolerates failure without retaliation (Schein & Bennis, 1965) and the ability to “employ one's self without fear of negative consequences to self‐image, status, or career” (Kahn, 1990). This means that team members are encouraged to try things out and to stand up for their opinions and ideas, and do not have to be afraid of being demoted as a result. Later, also by drawing on evidence from health‐care provision, Edmondson defines psychological safety at the group level “as a shared belief that the team is safe for interpersonal risk‐taking”—being safe for interpersonal risk‐taking means that nurses do not fear punishment when speaking up (Edmondson, 1999). Furthermore, team members who feel psychologically safe also feel that their skills and talents are valued and mistakes are not held against them (Nembhard & Edmondson, 2006). What all these definitions have in common is the belief that psychological safety facilitates the willing contribution of ideas and actions to a shared enterprise (Edmondson & Lei, 2014).

There are three different aspects why psychological safety is important in the context of patient care. First, employees who feel psychologically safe dare to speak up and raise their concerns, for example about an erroneously prescribed medical dosage (Bienefeld & Grote, 2014; Edmondson & Lei, 2014). By addressing their concerns, possible medical failures and treatment errors in general can be avoided and thus lead to a higher patient safety. This is particularly important in intensive care units, where the severity of the consequences of errors due to complex medical processes and vulnerable patients can be particularly high. This is the main reason why we have chosen to focus on nurses in neonatal intensive care units (NICUs). NICUs are used to treat patients who are seriously ill and sensitive to disruptions (Profit et al., 2012). Second, previous research has established that psychological safety fosters employee engagement, commitment, and job satisfaction and reduces bullying (Arnetz et al., 2019; Edmondson, 2003; Frazier et al., 2017; May et al., 2004). Nowadays, when it is important to keep good nursing staff, these aspects are crucial outcomes for hospitals. Third, psychological safety enables team members to modify and apply new technologies (Edmondson et al., 2000) and foster learning behaviour (Edmondson, 2003; Ortega et al., 2013); it is also important for information exchange amongst team members (Aranzamendez et al., 2015).

To date, various factors at an individual and an organizational level are known to affect psychological safety. Prior research has established that leader and team support are important antecedents of psychological safety. People feel their contributions are valued if they receive support from their leader and team (Schepers et al., 2008). The significance of the leader is also figured out in the meta‐analytic review of Frazier et al. They outlined, for example, a positive association between psychological safety and ethical leadership, servant leadership, leader–member exchange and trust in one's leader (Frazier et al., 2017). Moreover, at the individual level the two *Big Five* personality constructs *emotional stability* (as the opposite of *neuroticism*) and *openness to experience* are theoretically linked to psychological safety. Persons with these characteristics “tend to be calm, relaxed, and secure as opposed to anxious, hostile, and vulnerable to stress” (Frazier et al., 2017). Frazier et al. also found a positive and significant relationship between a proactive personality and psychological safety (Frazier et al., 2017). Employees with a proactive personality find it easier to seek performance feedback and build social networks (Crant, 2000). Further, previous research suggests that professional experience in NICUs in general, and in the current NICU, is associated with higher psychological safety (Nembhard & Edmondson, 2006).

## 3 THEORETICAL FRAMEWORK AND DEVELOPMENT OF HYPOTHESES

Previous studies found professional status and hierarchical constraints as an antecedent of psychological safety and speaking up, respectively (for example, Morrow et al., 2016). In general, people of lower status undervalue their input and even fear negative consequences if they speak up (Bienefeld & Grote, 2014). In the health‐care context, Lyndon et al. report that nurses rate potential harm in common clinical scenarios more highly than physicians and are consequently less likely to speak up (Lyndon et al., 2012). Nembhard and Edmondson examined the effect of professional status on psychological safety. They revealed that physicians perceive higher psychological safety than nurses due to their higher professional standing (Nembhard & Edmondson, 2006). There has been less investigation into possible status differences amongst nurses, that is, *within* the profession, and the resulting effects on their individual perceptions of psychological safety.

The individual's status in a group depends on one's possession or embodiment of generally desired attributes or characteristics (Benoit‐Smullyan, 1944). In the professional context of nursing, additional training is a desired attribute that leads to higher individual status within the group. Wolff et al. suggest that nurses’ educational attainment is one of the most relevant attributes affecting their attitudes and behaviour (Wolff et al., 2010). Based on the status characteristic theory, if a nurse has completed additional training, her/his occupational prestige—and thus her/his status in the group—increases (Bloom, 1980). Prior research has shown that employees with a higher status are more likely to participate in team decisions (Bloom, 1980), to speak up (Bienefeld & Grote, 2014) and to feel more psychologically safe (Nembhard & Edmondson, 2006). Thus, we assume that nurses with additional training also feel more psychologically safe than nurses without additional training.

In our research, we differentiate between two types of additional training that focus on different professional aspects.

The additional *managerial* training course as a charge nurse enables nurses to undertake management and leadership tasks. Although these nurses are not the NICU’s leading nurse despite their advanced training, they are not inferior to the leading nurse in terms of education. Thus, we expect the status difference between these nurses and the leading nurse to be lower. Furthermore, nurses also learn soft skills during their additional training, such as communication and solution‐oriented responses to conflict situations. These skills are associated with a higher psychological safety due to a higher likelihood of the nurse in question speaking up (Landgren et al., 2016). These considerations lead to our first hypothesis:

Hypothesis 1: Nurses with an additional managerial training course as a charge nurse perceive higher psychological safety than nurses without an additional managerial training course as a charge nurse.

The two additional *clinical* training courses in paediatric intensive care and anaesthesia and intensive care enable nurses to perform core tasks in the care and support of (paediatric and) intensive care patients. Due to their additional clinical training, they possess more knowledge and are more likely to care for the patients in the most severe condition. Prior research has shown that the level of experience, the nurse's specialization and their confidence in their clinical knowledge are primary drivers for speaking‐up behaviour (Aiken & Sloane, 1997; Landgren et al., 2016; Lyndon et al., 2012). We differentiate between two different types of additional clinical training: “paediatric intensive care” and “anaesthesia and intensive care.” The former is more focussed on paediatric patients and therefore corresponds to the patient group in NICUs, whilst the latter is a more general type of training for nurses working in intensive care units, and does not focus on paediatric patients. Nevertheless, we expect there to be status differences between nurses with these types of additional clinical training and nurses without one of these types of additional clinical training. Thus, we hypothesize:

Hypothesis 2: Nurses with additional clinical training in paediatric intensive care perceive higher psychological safety than nurses without additional clinical training in paediatric intensive care.

Hypothesis 3: Nurses with additional clinical training in anaesthesia and intensive care perceive higher psychological safety than nurses without additional clinical training in anaesthesia and intensive care.

Beside these associations with the individual level of nurses, our second purpose is to focus on nurses with additional *clinical* training at the organizational level. Staffing guidelines for neonatal intensive care units require additional *clinical* training rates, that is, a legally fixed share of nurses has to show a certain level of education and specialization. As prior research has shown, team diversity, for example due to differences in education levels, can affect team communication and performance (for example, Bowers et al., 2000; Jackson, 1996). These differences in experience‐based status might limit the willingness of team members to communicate and interact with one another (Jackson et al. 1995). We aim to explore the share of NICU nurses who have additional clinical training as one indicator of team diversity. We assume that a high share of nurses with additional clinical training lowers the status differences for those who also completed additional clinical training. As more nurses possess additional clinical training, the team becomes more homogeneous, and additional clinical training becomes less likely to afford a nurse higher status within the team. Consequently, we expect the positive associations between a nurse's additional clinical training and the individually perceived psychological safety to be weakened then. Thus, our last hypothesis is:

Hypothesis 4: Nurses with additional clinical training do not perceive higher psychological safety if the NICU’s share of nurses with additional clinical training is high.

## 4 THE STUDY

### 4.1 Objective

We aim to derive management implications for nursing managers and nursing teachers, which allow safer designing of the work environment for nurses. Further, we finally intend to fill the research gap and react to the call of Nembhard and Edmondson to investigate the effect of nurses’ specialization on psychological safety (Nembhard & Edmondson, 2006).

### 4.2 Design

A multi‐level, multi‐source cross‐sectional survey called Safety4NICU study was conducted between September 2015 and August 2016 using data from 1,239 nurses and their corresponding leading nurses from 75 different NICUs in Germany.

### 4.3 Method

Two hundred and twenty‐four NICUs in Germany were identified by web‐based searches and public reports. Using a simple random sampling method, all of these were contacted by post and eligible to be included in the Safety4NICU study. The participation of a NICU required the written informed consent of both the leading nurse and the head physician. Overall, 86 NICUs agreed to participate in the study. Each leading nurse received a personalized questionnaire. Moreover, anonymous staff questionnaires and self‐addressed reply envelopes were sent to the leading nurse with the request to distribute them to all nursing staff at a team meeting. All staff nurses who worked at least 50% of a full‐time equivalent in the NICU were eligible to participate in the study. These nurses are most familiar with their NICU’s structure, procedures and teamwork.

Both the leader and the staff questionnaires were sent back to an independent operating data trust unit located at the University of Cologne. The data collection was carried out between September 2015 and August 2016. Within this period, data from each NICU were collected once. Overall, 78 leading nurses and 1,406 nurses from 82 different NICUs sent back their questionnaires.

### 4.4 Analysis

Due to the hierarchical structure of our data, we conducted three different random intercept models that are nested. Model 1 only includes variables on organizational level. Model 2 also includes our variables of interest on the individual level. Model 3 is based on Model 2 and includes two interaction terms that analyse the effect of a high share of nurses with additional clinical training on the associations between a nurse's additional clinical training and her/his psychological safety. StataSE 16 was used for statistical analyses.

**FIGURE A1:**
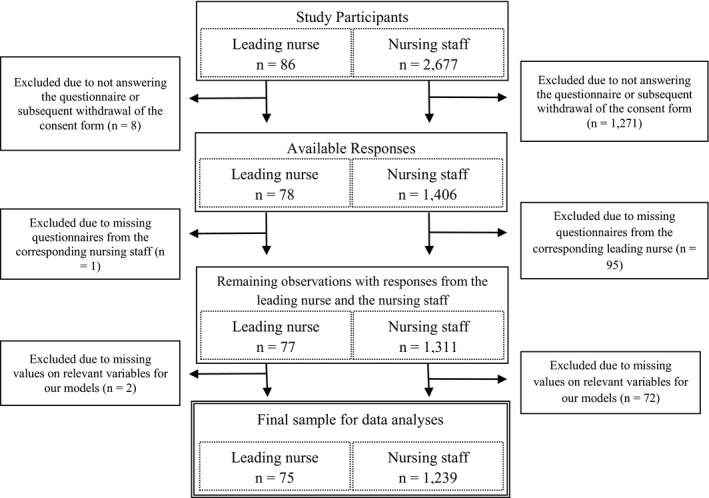
Sample rationale

Nevertheless, only teams whose response rate met the requirement of a sampling ratio of >.32, based on Dawson (2003) and Richter et al., (2006), were included in our analyses. One questionnaire of a leading nurse was excluded due to missing data of the corresponding nursing staff, whilst 95 questionnaires of nursing staff were excluded due to missing data of the corresponding leading nurse. Further, 72 questionnaires of nursing staff and two questionnaires of the leading nurses were excluded due to missing values on relevant variables for our models. Finally, we were able to match and analyse the data from 1,239 nurses in 75 NICUs (see Figure A1 in the Appendix).

### 4.5 Measurements

**Psychological safety**

Our dependent variable *psychological safety* was measured with four items on a 7‐point Likert scale (1 denoting complete disagreement; 7 denoting complete agreement), whereas higher scores indicate high psychological safety. The scale was adapted from Edmondson's psychological safety scale (Edmondson, 1999) and has already been applied in a NICU setting by Nembhard and Edmondson (2006). The scale was translated into German by in‐depth discussion with a bilingual German/English) psychologist with expertise in item translation. For example, nurses were asked whether the people in their NICU valued the unique skills and talents of others. A one‐factor confirmatory factor analysis (CFA) model showed good fit to the data (χ^2^ = 8.75, *df* = 2, *p* < .01, comparative fit index (CFI) = .995, Tucker‐Lewis index (TLI) = .986, root mean square error of approximation (RMSEA) = .052). The internal consistency of the scale was satisfactory (α = .76).

#### Additional training

Nurses were asked to state whether they had completed additional *managerial* training as a charge nurse and/or additional *clinical* training in paediatric intensive care and/or anaesthesia and intensive care.

#### High share of nurses with additional clinical training

We calculated the share of nurses by summing up all nurses in each NICU with additional clinical training, that is, paediatric intensive care and/or anaesthesia and intensive care, and dividing this sum by the number of respondents in each NICU. Using a median split to identify a high share of nurses in the NICU with additional clinical training, we generated a new dummy variable. NICUs whose share of nurses with additional clinical training was 46.67% or higher had a high share of nurses with additional clinical training.

### 4.6 Control variables

#### Job tenure

Nurses were asked to state their job tenure in their current NICU in years and months.

#### Leader tenure

The NICU’s leading nurse was asked to state her/his job tenure in her/his current position in years and months.

#### Collective team tenure

Nurses were asked to state their job tenure in their current NICU in years and months. Based on these data, we calculated the units’ mean and used this as a unit‐level measure for team tenure.

#### Perceptions of management

The perceptions of management within the team were measured by the Safety Attitude Questionnaire (SAQ) (Sexton et al., 2006) for each nurse. The SAQ dimension perceptions of management consist of four items on a 7‐point Likert scale (1 denoting complete disagreement; 7 denoting complete agreement), whereas higher scores indicate a high perception of management. We used a German version validated by Zimmermann et al., (2013) and slightly rephrased the items for our NICU setting (for example, “the leading nurse of this NICU supports my daily efforts”).

#### Team size

The NICU’s leading nurse was asked to state the number of nurses working in her/his NICU.

## 5 RESULTS

### 5.1 Respondent characteristics

Our final data set consists of responses from 1,239 nurses in 75 NICUs (see Table 1) and covers about one third of all German NICUs. The average response rate per team was 52.1% (*SD*: 0.182). 98.14% nurses were female. The average job tenure in the current NICU was 11.78 years. 5.08% of nurses had completed additional training as a charge nurse; 35.59% had additional training in paediatric intensive care; and 21.15% had additional training in anaesthesia and intensive care. The average rating on individually perceived psychological safety was 4.94 on a 7‐point Likert Scale.

**TABLE 1:** Participants' characteristics

	Valid	*n* (%)	Mean (*SD*)
Nursing staff (*n* = 1,239)	‐	‐	‐
Gender (female)	1,237	1,214 (98.14)	‐
Age	1,237	‐	‐
≤24 years	‐	125 (10.11)	‐
25–34 years	‐	400 (32.34)	‐
35–44 years	‐	296 (23.93)	‐
45–54 years	‐	324 (26.19)	‐
55–64 years	‐	92 (7.44)	‐
Job tenure (years)	1,239	‐	11.78 (10.26)
Additional training	1,239	‐	‐
Charge nurse	‐	63 (5.08)	‐
Paediatric intensive care	‐	441 (35.59)	‐
Anaesthesia and intensive care	‐	262 (21.15)	‐
Psychological safety	1,239	‐	4.94 (1.17)
NICU (*n* = 75)	‐	‐	‐
Leader tenure (years)	75	‐	10.89 (8.92)
Collective team tenure (years)	75	‐	10.27 (5.68)
SAQ: Perceptions of management	75	‐	.349 (.221)
Team size	75	‐	34.31 (12.45)
High share of nurses with additional clinical training	75	37 (49.33)	‐

Abbreviations: NICU: Neonatal intensive care unit; SAQ: Safety Attitude Questionnaire.

### 5.2 Analyses

We tested for multicollinearity by examining the Pearson correlation coefficients (*r*) between all relevant variables (see Table A1 in the Appendix). As there is no correlation coefficient between the independent variables higher than .4, we do not expect multicollinearity.

The null model shows an intraclass correlation coefficient of .1941, which means that 19.41% of all variance about the individually perceived psychological safety can be explained by NICU membership. Table 2 provides the results of the first two multi‐level models, which investigate the associations between organizational and individual factors on the nurses’ individually perceived psychological safety. Model 1 shows that the leader tenure and a high level of positive responses towards the perceptions of management are positively associated with individually perceived psychological safety (β = .144, 95% CI:.034–.253, *p* ≤ .01 and β = .315, 95% CI: .210–.420, *p* ≤ .001). Model 2 reveals that, at the individual level, the additional managerial training as a charge nurse is significantly positively related to a nurse's psychological safety (β = .346, 95% CI: .070–.622, *p* ≤ .05). Therefore, Hypothesis 1, which posited a positive relationship between additional managerial training as charge nurse and psychological safety, is supported. However, Hypothesis 2, which posited a positive relationship between additional clinical training in paediatric intensive care and psychological safety, could not be supported. We even found that additional clinical training in paediatric intensive care is negatively associated with psychological safety (β = −.192, 95% CI: −.325 to −.060, *p* ≤ .01). Hypothesis 3, which posited a positive association between additional clinical training in anaesthesia and intensive care and a nurse's psychological safety, could not be supported either. We did not find any significant association between these two variables. We also tested the association between psychological safety and two completed additional clinical training courses, that is, paediatric intensive care and anaesthesia and intensive care. Nonetheless, there was no significant association.

**TABLE 2:** Results of model 1 and model 2

	Model 1		Model 2	
	*β*	*p*	*β*	*p*
Nursing staff				
Job tenure	‐	‐	.030	.343
Advanced training	‐	‐	‐	‐
Charge nurse	^‐^	‐	.346^*^	.014
Paediatric intensive care	‐	‐	−.192^**^	.005
Anaesthesia and intensive care	‐	‐	.033	.676
NICU				
Leader tenure	.144^**^	.010	.146^**^	.010
Collective team tenure	−.031	.570	−.039	.494
SAQ: Perceptions of management	.315^***^	.000	.312^***^	.000
Team size	−.080	.138	−.071	.196
High share of nurses with additional clinical training	.075	.492	.105	.350
*N*				
Observations	1,239	‐	1,239	‐
Groups	75	‐	75	‐
Log likelihood	−1,865.92	‐	−1,858.67	‐
ICC	.1086	‐	.1157	‐
AIC	3,747.85	‐	3,741.33	‐

Note

^*^ *p* ≤.05, **p ≤.01, ***p ≤.001.

Abbreviations: AIC: Akaike Information Criterion; ICC: Intra‐class Correlation Coefficient; NICU: Neonatal intensive care unit; SAQ: Safety Attitude Questionnaire.

Further, we analysed the effects of additional clinical training in paediatric intensive care and anaesthesia and intensive care on psychological safety, respectively, depending on the NICU’s share of nurses with additional clinical training.

**FIGURE 1:**
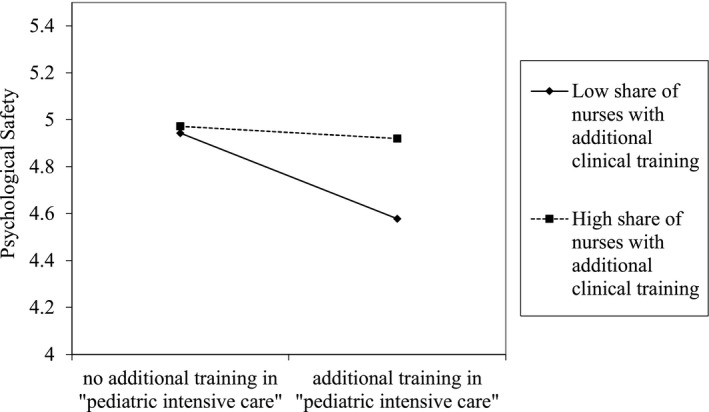
Interaction effect of model 3

The results in Table 3 demonstrate that, for nurses with additional clinical training in paediatric intensive care, the share of nurses with additional clinical training is highly relevant and even leads to a significantly positive association between the additional clinical training in paediatric intensive care and individual psychological safety. Figure 1 shows that, for nurses with additional training in paediatric intensive care, a high share of nurses with additional clinical training prevents them from feeling less psychologically safe. This effect was not found for nurses with the additional training in anaesthesia and intensive care. Thus, Hypothesis 4, which posited that nurses with additional clinical training do not perceive higher psychological safety if the NICU’s share of nurses with additional clinical training is high, is partially supported.

**TABLE 3:** Results of model 3

	Model 3	
	β	*p*
Nursing Staff		
Job tenure	.028	.375
Advanced training	‐	‐
Charge nurse	.343^*^	.015
Paediatric intensive care	−.365^***^	.000
Anaesthesia and intensive care	.138	.261
Paediatric intensive care & high share of nurses with additional clinical training	.313^*^	.021
Anaesthesia and intensive care & high share of nurses with additional clinical training	−.157	.332
NICU		
Leader tenure	.143^*^	.012
Collective team tenure	−.038	.504
SAQ: Perceptions of management	.310^***^	.000
Team size	−.076	.167
High share of nurses with additional clinical training	.029	.818
*N*		
Observations	1,239	‐
Groups	75	‐
Log likelihood	−1,855.90	‐
ICC	.1187	‐
AIC	3,739.81	‐

Note

^*^ *p* ≤.05, ^**^ *p* ≤.01, ^***^ *p* ≤.001.

Abbreviations: AIC: Akaike Information Criterion; ICC: Intra‐class Correlation Coefficient; NICU: Neonatal intensive care unit; SAQ: Safety Attitude Questionnaire.

## 6 DISCUSSION

The objectives of our study were to examine the associations between a nurse's psychological safety and her/his additional training, and investigating the moderating effect of a NICU’s share of nurses with additional clinical training. Our data show an individually perceived psychological safety level of 4.94. This average value is slightly lower than the reported value of 5.31 perceived by nurses, physicians, respiratory therapists and other health‐care professionals in NICUs (Nembhard & Edmondson, 2006), but is higher than the reported value of 3.50 perceived by nurses in public hospitals (Ortega et al., 2013).

The most striking result to emerge from the data is that the associations between psychological safety and the three various additional types of training are different.

First, the additional managerial training as a charge nurse is significantly positively associated with a nurse's individually perceived psychological safety. Although these nurses with the additional managerial training are not their NICU’s leading nurse, they feel more psychologically safe than nurses without this managerial training. Therefore, the reasons for this positive association might be based primarily on their acquired skills and their associated standing in the team. There are two likely causes for this positive association. On the one hand, leadership skills and other managerial issues that nurses have learnt during their additional managerial training could empower nurses to speak up (Landgren et al., 2016). For example, part of this additional managerial training focuses on acting as a leader, communication skills and the right way to deal with conflicts. These nurses may find it easier to address conflict situations and unclear procedures, which in turn increases their psychological safety (Erkutlu & Chafra, 2015). On the other hand, managerial training might reduce the status differences between these nurses and the other superiors on the NICU, especially the leading nurse, as there is little to no difference in their respective educational levels.

Second, the association between the psychological safety and the additional clinical training in paediatric intensive care is significantly negative. This result highlights that the additional training according to the working environment does not immediately lead to increased psychological safety. On the contrary, the finding suggests that these nurses feel less psychologically safe. This result might be explained by possible difficulties for these nurses to implement their knowledge and expertise. As a result, these nurses feel that their skills and expertise are not valued, and consequently feel less psychologically safe (Nembhard & Edmondson, 2006). For example, possible treatment options that are proposed by these nurses might be viewed sceptically by nurses without a clinical training. This explanation is consistent with the findings of Kvarnström (2008), which indicated that a lack of consensus is a typical difficulty in teamwork when new and unknown skills are added to the team (Kvarnström, 2008). The moderating positive effect of a high share of nurses with additional clinical training underlines this assumption. If the share of nurses with additional clinical training is high, nurses with the additional clinical training in paediatric intensive care feel more psychologically safe. In NICUs where a high share of nurses possess additional clinical training, it might be easier to implement expertise and experience peer support due to the relatively even distribution of up‐to‐date knowledge (Kvarnström, 2008). There are also two further explanations for this result. Firstly, prior research has shown that individuals of similar background and abilities are attracted to one another (Bowers et al., 2000). It is easier and more desirable for them to interact (Williams Phillips & O'Reilly, 1998). It can be expected that nurses with the same educational background have high‐level relationships based primarily on their shared knowledge, which increase their psychological safety (Carmeli & Gittell, 2009). This is also confirmed by research on person–environment fit, which shows that there is a positive association between the supplementary fit, that is, the similarities between a person and her/his team members, and their psychological safety (Cooman et al., 2016; Seong et al., 2015; Stonefish, 2019). Secondly, if we link this result to social network theory, a further explanation might be that a person feels more psychologically safe the more so‐called *advice ties* she or he receives (Schulte et al., 2012). This means that nurses with the specialized additional training in paediatric intensive care might receive less advice and support from other nurses in their NICU, as there are not many other nurses whose level of expertise is equal to or higher than their own. The probability that these nurses receive advice and support may increase if the share of nurses with additional clinical training is high, which in turn increases their psychological safety once more (Schepers et al., 2008; Schulte et al., 2012).

Third, the additional clinical training in anaesthesia and intensive care is not associated with a nurse's psychological safety. The extent and the content of this training are similar to those of the additional clinical training in paediatric intensive care, but do not focus on paediatric patients. As their degree of specialization about paediatric patients is not as high as the degree of nurses with additional clinical training in paediatric intensive care, situations in which these nurses try to implement specialized expertise and meet with incomprehension or even criticism may possibly arise less often. Thus, these nurses might be less likely to feel that their skills and expertise are not valued, which is one typical characteristic of psychological safety (Edmondson, 1999). Compared to nurses with additional clinical training in paediatric intensive care, nurses with additional clinical training in anaesthesia and intensive care may be more likely to experience peer support and advice from nurses who are more specialized. These support and advice increase their sense of psychological safety (Schulte et al., 2012).

Additionally, our control variables emphasize the importance of the NICU’s leading nurse to promote psychological safety. As confirmed by the literature, there is a positive association between positive responses towards the perceptions of management figures (which cover factors such as a leader's support and the way he/she deals with problematic staff) and a nurse's psychological safety (Aranzamendez et al., 2015; Edmondson & Roloff, 2009; Frazier et al., 2017). The positive association between a leader's job tenure in her/his current position and a nurse's psychological safety can be explained by the level of comfort nurses feel when talking to a leader who they have known as their leader for a long time (O'Donovan et al., 2021).

## 7 IMPLICATIONS

For nurses with additional clinical training in paediatric intensive care, we found that a high share of nurses with additional clinical training can be advantageous to enhancing their psychological safety. It might be important that these nurses with specialized knowledge and skills receive support, understanding and advice from the team. Our results show that it is not sufficient to impart specialist knowledge; the curriculum of additional clinical training should also contain lessons on how to deal with concerns and (near) failures. More research will need to be done to investigate the negative effects of additional clinical training on psychological safety to infer possible actions that could be taken in order to control unintended negative effects of additional clinical training.

## 8 LIMITATIONS

There are some limitations to our study. We measured the nurses’ psychological safety at one point in time. It is still unclear whether nurses with additional clinical training in paediatric intensive care, for example, had perceived an even lower psychological safety level before completing the additional training. Moreover, we did not investigate whether those nurses who had completed additional managerial training as charge nurses felt psychologically safe before and therefore asked for additional managerial training as charge nurses, or else felt psychologically safe after completing additional training. Moreover, nurses with additional managerial training as charge nurses may hold a leadership position (not that of the leading nurse in our sample) and thus may not assess their psychological safety neutrally, as they fear this could devaluate their leadership skills. Further, as we focussed on intensive and paediatric care, the generalizability of our findings might be limited. Thus, further research should measure the nurses’ psychological safety level at different points in their careers and should also investigate other additional training and associations with psychological safety.

## 9 CONCLUSION

The current study provides new insights into the professional education and specialization in nursing about psychological safety.

The nurses’ additional training is associated with psychological safety in different ways. We identified two different aspects in our study. On the one hand, additional *managerial* training that focuses on management issues and leadership skills is positively associated with psychological safety. On the other hand, additional *clinical* training that focuses on specialized care and medical knowledge (in this context, paediatric) is negatively associated with psychological safety. A further analysis showed that this negative association could be inhibited if the share of nurses with additional clinical training is high. We assume that it is easier for nurses with additional clinical training in paediatric intensive care to implement their knowledge and expertise then. They might experience less incomprehension and criticism. In promoting psychological safety, and thus designing a work environment that enables nurses to speak up and to feel safe, our new insights appeal for an increase in the share of nurses with specialized knowledge in order to achieve a certain degree of supplementary fit between individual nurses with specialized knowledge and their team members. Furthermore, our results suggest adapting the learning content of additional clinical training.

## 10 RESEARCH ETHICS COMMITTEE APPROVAL

This article draws on data from the cross‐sectional survey—the Safety4NICU study. Amongst 86 participating NICUs, the nurses and physicians were asked to fill out pseudonymized questionnaires. All participants were informed that participation was voluntary, anonymous and do not have any impact on their employment. An ethics approval was granted by the Ethics Committee of the University of Cologne, Germany and registered with the German Clinical Trials Register (DRKS) under the number DRKS00007724.

## CONFLICT OF INTEREST

The authors declare that there is no conflict of interest that could be perceived as prejudicing the impartiality of the research reported.

## Data Availability

Data sharing is not applicable to this article as participants of this study did not agree for their data to be shared publicly, so supporting data is not available.

## 1

**TABLE A1:** Pearson correlation coefficients (*r*)

Variables	(1)	(2)	(3)	(4)	(5)	(6)	(7)	(8)	(9)	(10)
(1) Psychological safety	1.000	‐	‐	‐	‐	‐	‐	‐	‐	‐
(2) Job tenure	.044 (.124)	1.000	‐	‐	‐	‐	‐	‐	‐	‐
(3) Additional training as charge nurse	.059* (.037)	.131* (.000)	1.000	‐	‐	‐	‐	‐	‐	‐
(4) Additional training in paediatric intensive care	−.056* (.050)	.045 (.117)	.089* (.002)	1.000	‐	‐	‐	‐	‐	‐
(5) Additional training in anaesthesia and intensive care	−.031 (.269)	.023 (.422)	.033 (.244)	.176* (.000)	1.000	‐	‐	‐	‐	‐
(6) Leader tenure	.120* (.000)	.075* (.008)	.013 (.646)	.070* (.013)	−.014 (.612)	1.000	‐	‐	‐	‐
(7) Collective team tenure	.043 (.134)	.323* (.000)	.047 (.102)	.068* (.017)	−.032 (.259)	.312* (.000)	1.000	‐	‐	‐
(8) Perceptions of management	.268* (.000)	.066* (.021)	.049 (.086)	−.013 (.659)	−.067* (.019)	.008 (.791)	.121* (.000)	1.000	‐	‐
(9) Team size	−.099* (.001)	−.021 (.453)	−.005 (.858)	.103* (.000)	.018 (.532)	.048 (.093)	−.017 (.544)	−.176* (.000)	1.000	‐
(10) High share of nurses with additional clinical training	.015 (.591)	.057* (.044)	.019 (.499)	.221* (.000)	.138* (.000)	.165* (.000)	.095* (.001)	−.094* (.001)	.074* (.009)	1.000

Note

****p* <.01, ***p* <.05, **p* <.1
